# 5-Amino-7-(3-chloro­phen­yl)-3,7-di­hydro-2*H*-thieno[3,2-*b*]pyran-6-carbo­nitrile 1,1-dioxide

**DOI:** 10.1107/S1600536809055202

**Published:** 2010-01-09

**Authors:** Shi-De Shen, Xiao-Dong Feng, Wei-Hua Yang, Cui-Hua Wang, Chang-Sheng Yao

**Affiliations:** a Xuzhou Institute of Architectural Technology, Xuzhou 221116, People’s Republic of China; b School of Chemistry and Chemical Engineering, Xuzhou Normal University, Xuzhou 221116, People’s Republic of China; c Key Laboratory of Biotechnology for Medicinal Plants, Xuzhou Normal University, Xuzhou 221116, People’s Republic of China

## Abstract

The title compound, C_14_H_11_ClN_2_O_3_S, with fused thiophene and pyran rings, was synthesized *via* the condensation of dihydro­thio­phen-3(2*H*)-one 1,1-dioxide and 2-(3-chloro­benz­yl­idene)malononitrile catalysed by triethyl­amine in ethanol. The thio­phene ring adopts an envelope conformation and the pyran ring is planar (r.m.s. deviation = 0.0067 Å). The dihedral angle between the pyran and phenyl rings is 80.8 (1)°. The crystal packing is stabilized by inter­molecular N—H⋯N and N—H⋯O hydrogen bonds in which the cyano N and sulphone O atoms, respectively, acting as acceptors.

## Related literature

For the use of thienopyranyl compounds, such as thieno[3,2-*b*]pyran derivatives, as anti­viral agents, see: Friary *et al.* (1991 ▶) and as α-2C adrenoreceptor agonists, see: Chao *et al.* (2009 ▶). For puckering parameters, see: Cremer & Pople (1975 ▶).
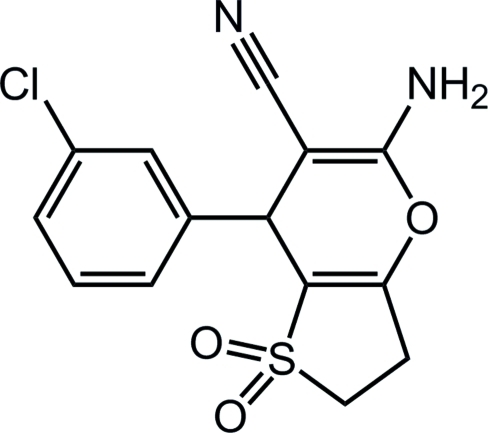


## Experimental

### 

#### Crystal data

C_14_H_11_ClN_2_O_3_S
*M* *_r_* = 322.76
Monoclinic, 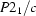

*a* = 9.5802 (19) Å
*b* = 17.364 (4) Å
*c* = 8.2521 (17) Å
β = 97.83 (3)°
*V* = 1360.0 (5) Å^3^
*Z* = 4
Mo *K*α radiation
μ = 0.45 mm^−1^
*T* = 113 K
0.20 × 0.18 × 0.12 mm

#### Data collection

Rigaku Saturn CCD area-detector diffractometer
Absorption correction: multi-scan (*CrystalClear*; Rigaku/MSC, 2005 ▶) *T*_min_ = 0.916, *T*_max_ = 0.949
9115 measured reflections
2393 independent reflections
1705 reflections with *I* > 2σ(*I*)
*R*_int_ = 0.096

#### Refinement

*R*[*F* ^2^ > 2σ(*F* ^2^)] = 0.053
*wR*(*F* ^2^) = 0.134
*S* = 1.08
2393 reflections
191 parameters
H-atom parameters constrained
Δρ_max_ = 0.67 e Å^−3^
Δρ_min_ = −0.49 e Å^−3^

### 

Data collection: *CrystalClear* (Rigaku/MSC, 2005 ▶); cell refinement: *CrystalClear*; data reduction: *CrystalClear*; program(s) used to solve structure: *SHELXS97* (Sheldrick, 2008 ▶); program(s) used to refine structure: *SHELXL97* (Sheldrick, 2008 ▶); molecular graphics: *SHELXTL* (Sheldrick, 2008 ▶); software used to prepare material for publication: *SHELXTL*.

## Supplementary Material

Crystal structure: contains datablocks I, New_Global_Publ_Block. DOI: 10.1107/S1600536809055202/hg2619sup1.cif

Structure factors: contains datablocks I. DOI: 10.1107/S1600536809055202/hg2619Isup2.hkl

Additional supplementary materials:  crystallographic information; 3D view; checkCIF report

## Figures and Tables

**Table 1:** Hydrogen-bond geometry (Å, °)

*D*—H⋯*A*	*D*—H	H⋯*A*	*D*⋯*A*	*D*—H⋯*A*
N2—H2*C*⋯N1^i^	0.88	2.20	3.060 (4)	165
N2—H2*D*⋯O1^ii^	0.88	2.04	2.912 (4)	174

Symmetry codes: (i) 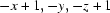; (ii) 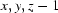.

**Fig. 1.**
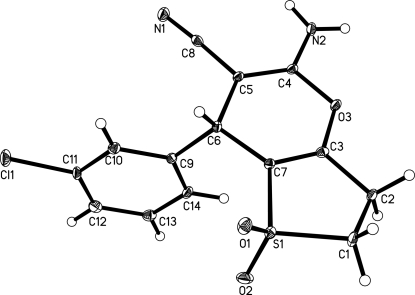
The structure of (I), showing 30% probability displacement ellipsoids and the atom-numbering scheme.

**Fig. 2.**
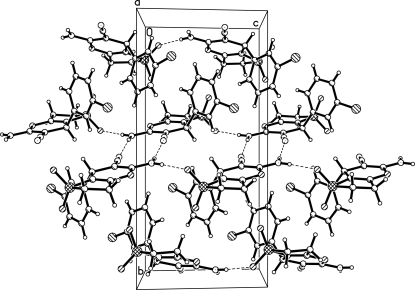
The packing diagram of (I). Intermolecular hydrogen bonds are shown as dashed lines.

## supplementary crystallographic information

### Comment

Thienopyranyl compounds, such as thieno [3,2-*b*]pyran derivatives, can be uesed as antiviral agents (Friary *et al.*, 1991) and α-2 C adrenoreceptor agonists (Chao *et al.*, 2009). This led us to pay attention to the synthesis and bioactivity of these compounds. During the synthesis of thieno[3,2-*b*]pyran derivatives, the title compound, (I) was isolated and its structure was determined by X-ray diffraction. Here we report its crystal structure.

The molecular structure of (I) is shown in Fig. 1. In the molecular structure, the thiophene ring is in envelope comformation, for the deviation of C1 from the C2/C3/C7/S1 plane is 0.354 (4)Å with r.m.s. of 0.010. The pyrane ring adopts a planar conformation. Cremer & Pople puckering analysis can not be performed, for its weighted average *ABS*. torsion angle is 1.0°, less than 5.0°. The connection of the pyrane ring and phenyl ring C9—C14 can be described by the C5—C6—C9—C14 torsion angle of 78.3 (3)°. The crystal packing is stabilized by intermolecular hydrogen bonds: N2—H2C···N1, N2—H2D···O1 (Fig.2 & Table 1).

### Experimental

The title compound was synthesized by the reaction of dihydrothiophen-3(2*H*)-one-1,1-dioxide (1 mmol) and 2-(3-chloro benzylidene)malononitrile (1 mmol) catalyzed by triethylamine (0.02 g) in 10 ml ethanol under reluxing until completion (monitored by TLC). Cooling the reaction mixture slowly gave single crystals suitable for X-ray diffraction.

### Refinement

All H atoms were placed in calculated positions, with N–H = 0.88 and C—H = 0.95, 0.99 or 1.00 Å, and included in the final cycles of refinement using a riding model, with *U*_iso_(H) = 1.2*U*_eq_(parent atom).

### Crystal data

**Table:**

C_14_H_11_ClN_2_O_3_S	*F*(000) = 664
*M**_r_* = 322.76	*D*_x_ = 1.576 Mg m^−^^3^
Monoclinic, *P*2_1_/*c*	Mo *K*α radiation, λ = 0.71073 Å
Hall symbol: -P 2ybc	Cell parameters from 4484 reflections
*a* = 9.5802 (19) Å	θ = 2.2–27.9°
*b* = 17.364 (4) Å	µ = 0.45 mm^−^^1^
*c* = 8.2521 (17) Å	*T* = 113 K
β = 97.83 (3)°	Block, colorless
*V* = 1360.0 (5) Å^3^	0.20 × 0.18 × 0.12 mm
*Z* = 4	

### Data collection

**Table:**

Rigaku Saturn CCD area-detector diffractometer	2393 independent reflections
Radiation source: rotating anode	1705 reflections with *I* > 2σ(*I*)
confocal	*R*_int_ = 0.096
Detector resolution: 7.31 pixels mm^-1^	θ_max_ = 25.0°, θ_min_ = 2.2°
ω and φ scans	*h* = −11→11
Absorption correction: multi-scan *CrystalClear*	*k* = −19→20
*T*_min_ = 0.916, *T*_max_ = 0.949	*l* = −9→9
9115 measured reflections	

### Refinement

**Table:**

Refinement on *F*^2^	Secondary atom site location: difference Fourier map
Least-squares matrix: full	Hydrogen site location: inferred from neighbouring sites
*R*[*F*^2^ > 2σ(*F*^2^)] = 0.053	H-atom parameters constrained
*wR*(*F*^2^) = 0.134	*w* = 1/[σ^2^(*F*_o_^2^) + (0.0583*P*)^2^] where *P* = (*F*_o_^2^ + 2*F*_c_^2^)/3
*S* = 1.08	(Δ/σ)_max_ < 0.001
2393 reflections	Δρ_max_ = 0.67 e Å^−^^3^
191 parameters	Δρ_min_ = −0.49 e Å^−^^3^
0 restraints	Extinction correction: *SHELXL*, Fc^*^=kFc[1+0.001xFc^2^λ^3^/sin(2θ)]^-1/4^
Primary atom site location: structure-invariant direct methods	Extinction coefficient: 0.491 (16)

### Special details

**Table:**

Geometry. All e.s.d.'s (except the e.s.d. in the dihedral angle between two l.s. planes) are estimated using the full covariance matrix. The cell e.s.d.'s are taken into account individually in the estimation of e.s.d.'s in distances, angles and torsion angles; correlations between e.s.d.'s in cell parameters are only used when they are defined by crystal symmetry. An approximate (isotropic) treatment of cell e.s.d.'s is used for estimating e.s.d.'s involving l.s. planes.
Refinement. Refinement of *F*^2^ against ALL reflections. The weighted *R*-factor *wR* and goodness of fit *S* are based on *F*^2^, conventional *R*-factors *R* are based on *F*, with *F* set to zero for negative *F*^2^. The threshold expression of *F*^2^ > σ(*F*^2^) is used only for calculating *R*-factors(gt) *etc*. and is not relevant to the choice of reflections for refinement. *R*-factors based on *F*^2^ are statistically about twice as large as those based on *F*, and *R*- factors based on ALL data will be even larger.

### Fractional atomic coordinates and isotropic or equivalent isotropic displacement parameters (Å^2^)

**Table:**

	*x*	*y*	*z*	*U*_iso_*/*U*_eq_	
S1	−0.01194 (8)	0.13574 (4)	0.99300 (11)	0.0141 (3)	
Cl1	0.69314 (8)	0.15345 (5)	1.25118 (11)	0.0240 (3)	
O1	−0.0062 (2)	0.07472 (12)	1.1133 (3)	0.0189 (6)	
O2	0.0250 (2)	0.21172 (12)	1.0529 (3)	0.0220 (7)	
O3	0.0422 (2)	0.09320 (11)	0.5466 (3)	0.0143 (6)	
N2	0.2045 (3)	0.05206 (14)	0.3987 (3)	0.0173 (7)	
H2C	0.2884	0.0361	0.3817	0.021*	
H2D	0.1367	0.0569	0.3159	0.021*	
N1	0.5321 (3)	0.03147 (15)	0.6812 (4)	0.0206 (7)	
C1	−0.1785 (3)	0.13702 (17)	0.8638 (4)	0.0157 (8)	
H1A	−0.2326	0.0898	0.8802	0.019*	
H1B	−0.2346	0.1822	0.8892	0.019*	
C2	−0.1464 (3)	0.14131 (16)	0.6882 (4)	0.0131 (8)	
H2A	−0.2124	0.1085	0.6155	0.016*	
H2B	−0.1545	0.1950	0.6474	0.016*	
C3	0.0022 (3)	0.11258 (17)	0.6944 (4)	0.0128 (8)	
C4	0.1798 (3)	0.06909 (16)	0.5505 (4)	0.0118 (7)	
C5	0.2722 (3)	0.06488 (17)	0.6910 (4)	0.0119 (7)	
C6	0.2351 (3)	0.08436 (16)	0.8608 (4)	0.0119 (7)	
H6	0.2458	0.0370	0.9305	0.014*	
C7	0.0834 (3)	0.10851 (16)	0.8370 (4)	0.0102 (7)	
C8	0.4147 (3)	0.04538 (16)	0.6824 (4)	0.0138 (8)	
C9	0.3344 (3)	0.14675 (16)	0.9401 (4)	0.0126 (8)	
C10	0.4501 (3)	0.12565 (18)	1.0523 (4)	0.0144 (8)	
H10	0.4640	0.0733	1.0839	0.017*	
C11	0.5450 (3)	0.18159 (19)	1.1175 (4)	0.0153 (8)	
C12	0.5249 (3)	0.25793 (18)	1.0762 (4)	0.0183 (8)	
H12	0.5893	0.2960	1.1233	0.022*	
C13	0.4088 (3)	0.27859 (18)	0.9644 (4)	0.0168 (8)	
H13	0.3941	0.3312	0.9351	0.020*	
C14	0.3145 (3)	0.22355 (17)	0.8953 (4)	0.0154 (8)	
H14	0.2365	0.2382	0.8177	0.018*	

### Atomic displacement parameters (Å^2^)

**Table:**

	*U*^11^	*U*^22^	*U*^33^	*U*^12^	*U*^13^	*U*^23^
S1	0.0099 (5)	0.0182 (5)	0.0140 (6)	0.0013 (3)	0.0011 (4)	−0.0018 (3)
Cl1	0.0124 (5)	0.0361 (6)	0.0215 (6)	0.0006 (3)	−0.0051 (4)	−0.0020 (4)
O1	0.0173 (13)	0.0255 (13)	0.0136 (15)	0.0041 (9)	0.0015 (11)	0.0050 (10)
O2	0.0201 (13)	0.0191 (13)	0.0280 (17)	−0.0022 (9)	0.0073 (12)	−0.0113 (11)
O3	0.0094 (12)	0.0199 (13)	0.0136 (14)	0.0039 (9)	0.0018 (10)	−0.0007 (10)
N2	0.0100 (14)	0.0277 (16)	0.0137 (18)	0.0025 (12)	−0.0008 (13)	−0.0038 (13)
N1	0.0137 (16)	0.0291 (16)	0.0185 (19)	0.0042 (12)	0.0008 (13)	−0.0018 (14)
C1	0.0081 (17)	0.0228 (18)	0.015 (2)	0.0021 (12)	−0.0006 (15)	0.0022 (14)
C2	0.0086 (17)	0.0147 (17)	0.016 (2)	0.0026 (12)	0.0004 (15)	0.0006 (13)
C3	0.0128 (17)	0.0104 (16)	0.016 (2)	0.0000 (12)	0.0048 (15)	0.0000 (14)
C4	0.0095 (16)	0.0117 (16)	0.015 (2)	0.0008 (12)	0.0035 (15)	−0.0004 (14)
C5	0.0095 (17)	0.0144 (16)	0.012 (2)	0.0026 (12)	0.0027 (15)	−0.0003 (14)
C6	0.0106 (17)	0.0149 (16)	0.010 (2)	0.0024 (12)	−0.0004 (15)	−0.0002 (13)
C7	0.0081 (16)	0.0109 (15)	0.012 (2)	−0.0008 (12)	0.0028 (14)	−0.0003 (14)
C8	0.0182 (19)	0.0133 (17)	0.010 (2)	−0.0004 (13)	0.0006 (15)	−0.0002 (13)
C9	0.0072 (17)	0.0187 (17)	0.012 (2)	0.0004 (12)	0.0027 (15)	−0.0031 (14)
C10	0.0138 (18)	0.0163 (17)	0.014 (2)	0.0018 (13)	0.0032 (16)	0.0002 (14)
C11	0.0075 (16)	0.0268 (19)	0.011 (2)	0.0012 (13)	−0.0009 (15)	−0.0019 (15)
C12	0.0118 (18)	0.0242 (19)	0.020 (2)	−0.0045 (13)	0.0059 (16)	−0.0060 (15)
C13	0.0156 (18)	0.0167 (17)	0.019 (2)	−0.0010 (13)	0.0070 (16)	−0.0016 (15)
C14	0.0094 (16)	0.0205 (18)	0.016 (2)	0.0032 (13)	0.0017 (14)	0.0004 (15)

### Geometric parameters (Å, °)

**Table:**

S1—O2	1.436 (2)	C3—C7	1.322 (5)
S1—O1	1.448 (2)	C4—C5	1.361 (5)
S1—C7	1.742 (3)	C5—C8	1.418 (4)
S1—C1	1.794 (3)	C5—C6	1.529 (4)
Cl1—C11	1.745 (3)	C6—C7	1.499 (4)
O3—C3	1.369 (4)	C6—C9	1.529 (4)
O3—C4	1.379 (4)	C6—H6	1.0000
N2—C4	1.339 (4)	C9—C14	1.390 (4)
N2—H2C	0.8800	C9—C10	1.393 (5)
N2—H2D	0.8800	C10—C11	1.388 (4)
N1—C8	1.152 (4)	C10—H10	0.9500
C1—C2	1.523 (5)	C11—C12	1.376 (4)
C1—H1A	0.9900	C12—C13	1.392 (5)
C1—H1B	0.9900	C12—H12	0.9500
C2—C3	1.503 (4)	C13—C14	1.383 (4)
C2—H2A	0.9900	C13—H13	0.9500
C2—H2B	0.9900	C14—H14	0.9500
			
O2—S1—O1	116.86 (15)	C8—C5—C6	116.4 (3)
O2—S1—C7	111.96 (13)	C7—C6—C9	113.2 (2)
O1—S1—C7	109.44 (13)	C7—C6—C5	106.5 (3)
O2—S1—C1	110.54 (14)	C9—C6—C5	109.9 (2)
O1—S1—C1	111.37 (14)	C7—C6—H6	109.1
C7—S1—C1	94.45 (15)	C9—C6—H6	109.1
C3—O3—C4	115.9 (3)	C5—C6—H6	109.1
C4—N2—H2C	120.0	C3—C7—C6	125.1 (3)
C4—N2—H2D	120.0	C3—C7—S1	109.8 (2)
H2C—N2—H2D	120.0	C6—C7—S1	125.1 (3)
C2—C1—S1	106.7 (2)	N1—C8—C5	177.0 (4)
C2—C1—H1A	110.4	C14—C9—C10	119.8 (3)
S1—C1—H1A	110.4	C14—C9—C6	120.7 (3)
C2—C1—H1B	110.4	C10—C9—C6	119.4 (3)
S1—C1—H1B	110.4	C11—C10—C9	119.6 (3)
H1A—C1—H1B	108.6	C11—C10—H10	120.2
C3—C2—C1	105.3 (3)	C9—C10—H10	120.2
C3—C2—H2A	110.7	C12—C11—C10	121.1 (3)
C1—C2—H2A	110.7	C12—C11—Cl1	120.0 (3)
C3—C2—H2B	110.7	C10—C11—Cl1	118.9 (2)
C1—C2—H2B	110.7	C11—C12—C13	119.0 (3)
H2A—C2—H2B	108.8	C11—C12—H12	120.5
C7—C3—O3	125.3 (3)	C13—C12—H12	120.5
C7—C3—C2	119.2 (3)	C14—C13—C12	120.9 (3)
O3—C3—C2	115.5 (3)	C14—C13—H13	119.6
N2—C4—C5	127.5 (3)	C12—C13—H13	119.6
N2—C4—O3	109.6 (3)	C13—C14—C9	119.7 (3)
C5—C4—O3	123.0 (3)	C13—C14—H14	120.2
C4—C5—C8	119.2 (3)	C9—C14—H14	120.2
C4—C5—C6	124.3 (3)		
			
O2—S1—C1—C2	97.0 (2)	C9—C6—C7—S1	−60.0 (3)
O1—S1—C1—C2	−131.35 (19)	C5—C6—C7—S1	179.2 (2)
C7—S1—C1—C2	−18.5 (2)	O2—S1—C7—C3	−104.5 (2)
S1—C1—C2—C3	21.1 (3)	O1—S1—C7—C3	124.3 (2)
C4—O3—C3—C7	−1.3 (4)	C1—S1—C7—C3	9.8 (2)
C4—O3—C3—C2	178.0 (2)	O2—S1—C7—C6	75.3 (3)
C1—C2—C3—C7	−16.2 (4)	O1—S1—C7—C6	−55.9 (3)
C1—C2—C3—O3	164.4 (2)	C1—S1—C7—C6	−170.4 (3)
C3—O3—C4—N2	180.0 (2)	C4—C5—C8—N1	149 (7)
C3—O3—C4—C5	−0.4 (4)	C6—C5—C8—N1	−28 (7)
N2—C4—C5—C8	4.5 (5)	C7—C6—C9—C14	−40.6 (4)
O3—C4—C5—C8	−175.0 (2)	C5—C6—C9—C14	78.3 (3)
N2—C4—C5—C6	−179.1 (3)	C7—C6—C9—C10	142.6 (3)
O3—C4—C5—C6	1.4 (5)	C5—C6—C9—C10	−98.5 (3)
C4—C5—C6—C7	−0.6 (4)	C14—C9—C10—C11	−0.4 (5)
C8—C5—C6—C7	175.8 (3)	C6—C9—C10—C11	176.3 (3)
C4—C5—C6—C9	−123.5 (3)	C9—C10—C11—C12	1.6 (5)
C8—C5—C6—C9	52.9 (3)	C9—C10—C11—Cl1	−177.3 (2)
O3—C3—C7—C6	2.1 (5)	C10—C11—C12—C13	−1.4 (5)
C2—C3—C7—C6	−177.2 (3)	Cl1—C11—C12—C13	177.5 (2)
O3—C3—C7—S1	−178.1 (2)	C11—C12—C13—C14	0.1 (5)
C2—C3—C7—S1	2.6 (4)	C12—C13—C14—C9	1.1 (5)
C9—C6—C7—C3	119.8 (4)	C10—C9—C14—C13	−0.9 (4)
C5—C6—C7—C3	−1.0 (4)	C6—C9—C14—C13	−177.6 (3)

### Hydrogen-bond geometry (Å, °)

**Table:**

*D*—H···*A*	*D*—H	H···*A*	*D*···*A*	*D*—H···*A*
N2—H2C···N1^i^	0.88	2.20	3.060 (4)	165
N2—H2D···O1^ii^	0.88	2.04	2.912 (4)	174

Symmetry codes: (i) −*x*+1, −*y*, −*z*+1; (ii) *x*, *y*, *z*−1.

## References

[bb1] Chao, J. H., Zheng, J. Y. & Aslanian, R. G. (2009). WO Patent, No. 2009020578.

[bb2] Cremer, D. & Pople, J. A. (1975). *J. Am. Chem. Soc.* **97**, 1354–1358.

[bb3] Friary, R. J., Schwerdt, J. H. & Ganguly, A. K. (1991). US patent, No. 5034531.

[bb4] Rigaku/MSC (2005). *CrystalClear* Rigaku/MSC Inc., The Woodlands, Texas, USA.

[bb5] Sheldrick, G. M. (2008). *Acta Cryst.* A**64**, 112–122. 10.1107/S0108767307043930 18156677

